# Supplementary material for: Efficacy, safety and complications of autologous fat grafting to the eyelids and periorbital area: A systematic review and meta-analysis
Source: PLoS One. 2021 Apr 1;16(4):e0248505. doi: 10.1371/journal.pone.0248505 (PMC8016360; doi:10.1371/journal.pone.0248505)
Supplement: S1 File — (ZIP) [file pone.0248505.s001.zip › full search strategy and search terms.docx]

PubMed

**#1** ((((((((((fat grafting) OR (lipograft)) OR (lipoinjection)) OR (lipotransfer)) OR (fat transfer)) OR (fat transplant)) OR (lipostructure)) OR (lipofilling)) OR (fat injection)) OR (lipomodeling)) OR (fat transplantation) Sort by: Most Recent

**#2** (eyelid) OR (periocular) Sort by: Most Recent

**#3** #1 AND #2 423

Cochrane

(fat grafting OR lipograft OR lipoinjection OR lipotransfer OR fat transfer OR fat transplant OR lipostructure OR lipofilling OR fat injection OR lipomodeling OR fat transplantation) in All Text AND (eyelid OR periocular) in All Text - (Word variations have been searched) 13

The date ranges for the database searches (before November 20, 2020)
