# Supplementary material for: Efficacy, safety and complications of autologous fat grafting to the eyelids and periorbital area: A systematic review and meta-analysis
Source: PLoS One. 2021 Apr 1;16(4):e0248505. doi: 10.1371/journal.pone.0248505 (PMC8016360; doi:10.1371/journal.pone.0248505)
Supplement: S1 File — (ZIP) [file pone.0248505.s001.zip › LetPub - Certificate 2020.pdf]

Certificate of English Language Editing

Manuscript Title:

Efficacy, safety, and complications of autologous fat grafting to the eyelids and periorbital area: A systematic review and meta-analysis

Date of Revision:

November 3, 2020

Abstract:

Background: In recent years, autologous fat grafting (AFG), also known as fat transfer or lipofilling, has been widely performed for periorbital rejuvenation and defect correction, although the evidence regarding its efficacy is still lacking. Besides, with respect to the periorbital region, it is invariably the earliest appearance area of the facial aging phenomenon. Therefore, a systematic review and meta-analysis is needed to evaluate the efficacy and safety of this technique.

Methods: A literature search was performed in PubMed, Embase, and the Cochrane library databases on November 1, 2019, adhering to the PRISMA guidelines, to identify all relevant articles. Then, a data extraction and standardization process was performed to assess all outcome data. Ultimately, the data were assessed using a random effects regression model with comprehensive meta-analysis software. This protocol was registered in the Prospective Register of Systematic Reviews at the National Institute for Health Research (being assessed).

Results: Thirty-six studies consisting of four cohorts and 32 case series with a total of 3997 cases were included. The mean follow-up was 1.5 years. Meta-analysis revealed a relatively high satisfaction rate of 93.7% (95% CI, 89.6%–96.2%). Frequent complications in 3997 patients receiving AFG were edema, chemosis, and contour irregularity, with an overall complication rate of 7.7% (95% CI, 4.3%–13.3%).

This document certifies that the manuscript listed above was copy edited for proper English language at LetPub. All of our language editors are native English speakers with long-term experience in editing scientific and technical manuscripts. We are committed to leveling the playing field for researchers whose native language is not English.

- Neither the research content nor the authors' intended meaning were altered in any way during the editing process.
- Documents receiving this certification should be considered ready for publication where language issues are concerned. *However, the authors may accept or reject LetPub's suggestions and changes at their own discretion.*
- If you have any questions or concerns about this edited document, please contact us at support@letpub.com

LetPub
Your Manuscript Editor

LetPub is an author service brand owned and operated by Accdon LLC. Headquartered in the Boston area, we are a full-spectrum author services company with a large team of US-based certified language and scientific editors, ISO 17001 accredited translators, and professional scientific illustrators and animators. We advocate ethical publication practices and are an official member of the Committee on Publication Ethics (COPE).

For more information about our company, services, and partnership programs, please visit www.letpub.com.

© 2020 Accdon, LLC. All Rights Reserved. Tel: 1-781-202-9968 Email: info@accdon.com Address: 400 Fifth Ave, Suite 530, Waltham, MA 02451, United States
