# Supplementary material for: Efficacy, safety and complications of autologous fat grafting to the eyelids and periorbital area: A systematic review and meta-analysis
Source: PLoS One. 2021 Apr 1;16(4):e0248505. doi: 10.1371/journal.pone.0248505 (PMC8016360; doi:10.1371/journal.pone.0248505)
Supplement: S1 File — (ZIP) [file pone.0248505.s001.zip › title page.docx]

**Efficacy, safety and complications of autologous fat grafting to the eyelids and periorbital area: A systematic review and meta-analysis**

**Fan Yang^1^, Zhaohua Ji^2^, Liwei Peng^3^, Ting Fu^2^, Kun Liu^2^, Wenjie Dou^4^, Jing Li^1^, Yuejun Li^1^*, Yong Long^2^*, Weilu Zhang^2^***

**Authors’ affiliations:**

^1^ Department of Plastic Surgery and Burns, Tangdu Hospital, The Fourth Military Medical University, Shanxi, China,;

^2^ Department of Epidemiology, Ministry of Education Key Lab of Hazard Assessment and Control in Special Operational Environment, School of Public Health, The Fourth Military Medical University, Shanxi, China;

^3^ Department of Neurosurgery, Tangdu Hospital, The Fourth Military Medical University, Shanxi, China;

^4^ Department of Plastic Surgery, Xijing Hospital, The Fourth Military Medical University, Shanxi, China;

*Corresponding authors:

[zhangweilu@126.com](mailto:zhangweilu@126.com) (WZ)

[longyong71@163.com](mailto:longyong71@163.com) (YL)

[liyj@fmmu.edu.cn](mailto:liyj@fmmu.edu.cn) (YL)
