# Supplementary figures and images for: Efficacy, safety and complications of autologous fat grafting to the eyelids and periorbital area: A systematic review and meta-analysis
Source: PLoS One. 2021 Apr 1;16(4):e0248505. doi: 10.1371/journal.pone.0248505 (PMC8016360; doi:10.1371/journal.pone.0248505)

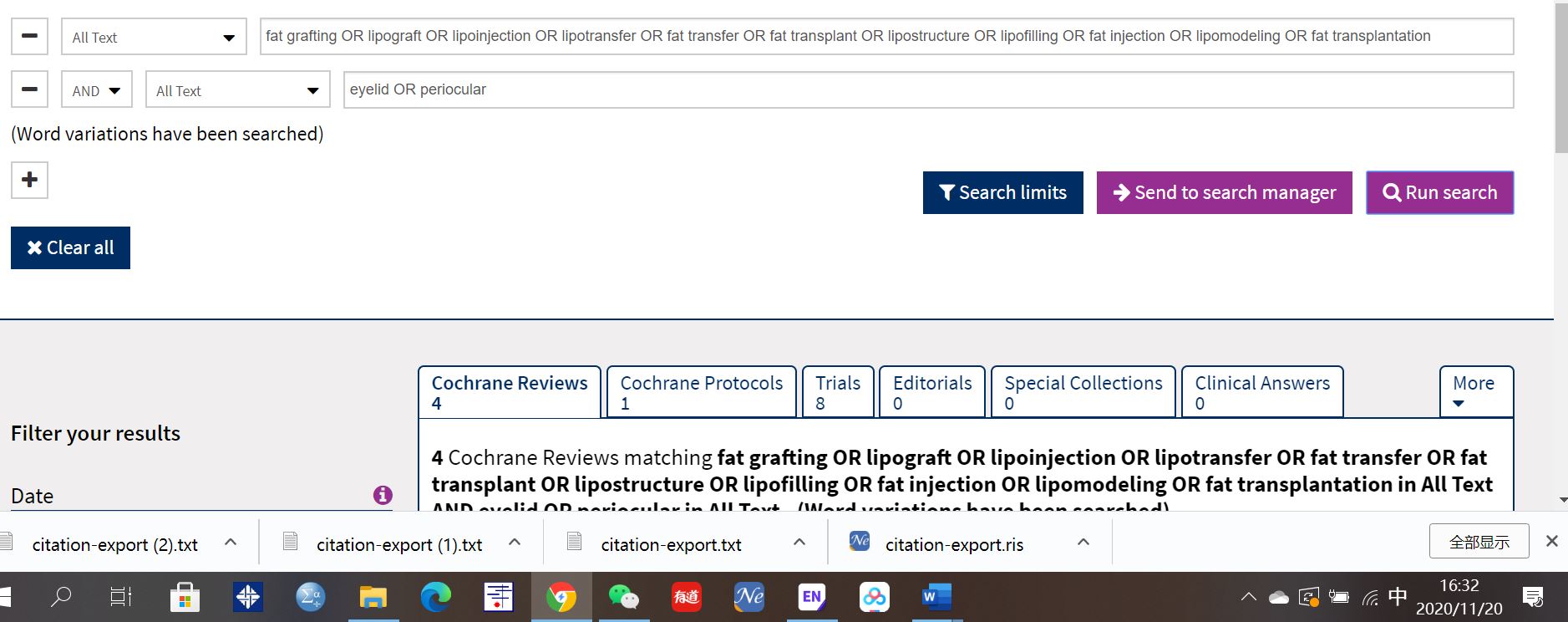

Supplement: S1 File — (ZIP) [file pone.0248505.s001.zip › original search image/cochrane library.JPG]

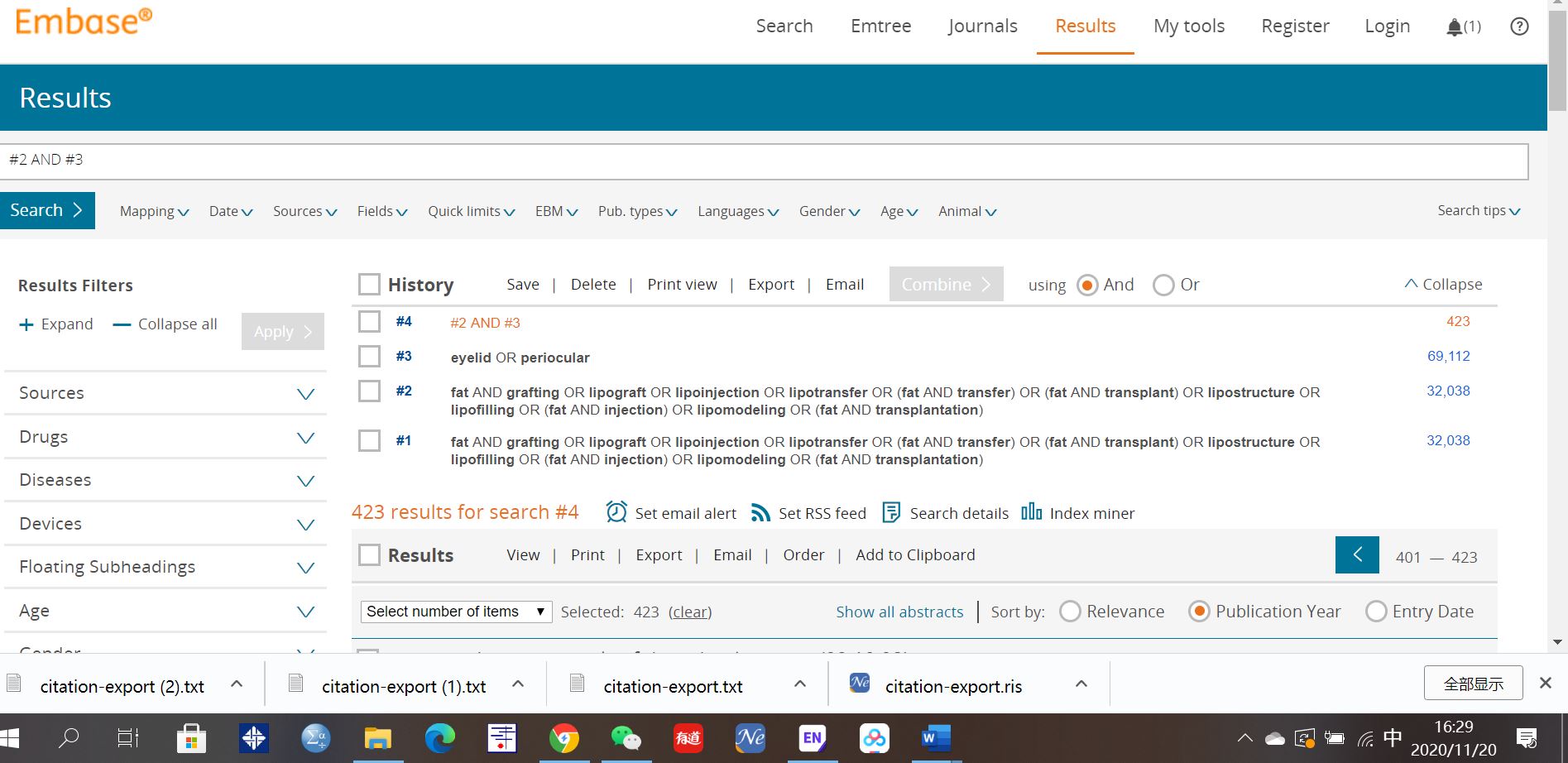

Supplement: S1 File — (ZIP) [file pone.0248505.s001.zip › original search image/embase.JPG]

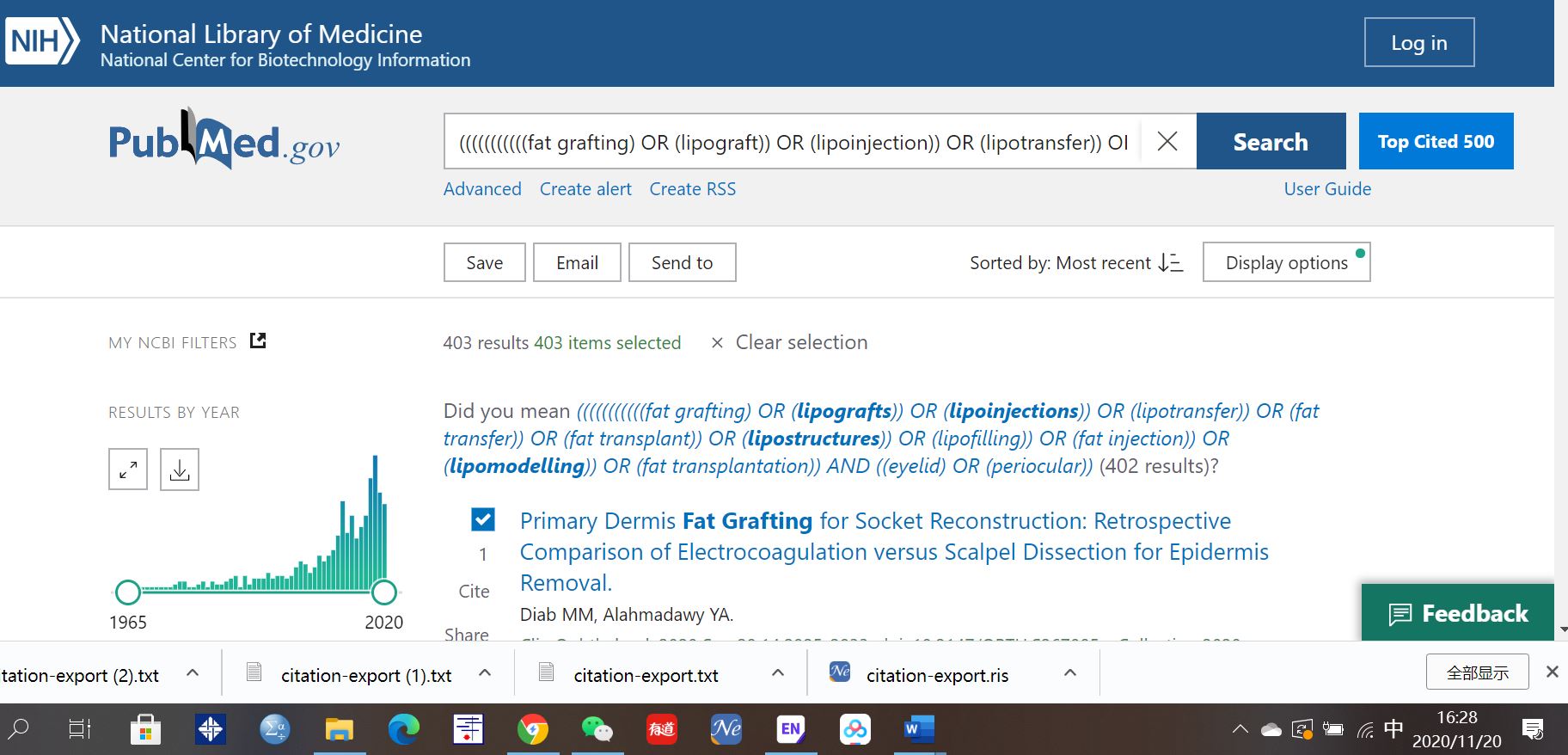

Supplement: S1 File — (ZIP) [file pone.0248505.s001.zip › original search image/PubMed.JPG]
