# Supplementary material for: Efficacy, safety and complications of autologous fat grafting to the eyelids and periorbital area: A systematic review and meta-analysis
Source: PLoS One. 2021 Apr 1;16(4):e0248505. doi: 10.1371/journal.pone.0248505 (PMC8016360; doi:10.1371/journal.pone.0248505)
Supplement: Attachment — Submitted filename: Response to Reviewers.docx [file pone.0248505.s002.docx]

Dear Editor,

We would like to express sincere gratitude for your letter and advice. We have revised the manuscript in accordance with the point raised by the academic editor and reviewers. Point-to-point answers have been provided below.

**Manuscript title:** Efficacy, safety and complications of autologous fat grafting for rejuvenation of eyelids and periorbital area: A systematic review and meta-analysis

**Academic editor**

1. *Please ensure that your manuscript meets PLOS ONE's style requirements, including those for file naming.*

Answer: Thank you for your comment. We have checked manuscript to meet the style requirements.

2. *We suggest you thoroughly copyedit your manuscript for language usage, spelling, and grammar. If you do not know anyone who can help you do this, you may wish to consider employing a professional scientific editing service.*

Answer: Thank you very much for your advice. We have sought help from the professional scientific editing service. Here is our Certi­ficate of English Language Editing.

3. *At this time, we ask that you please provide the full search strategy and search terms for at least one database used as Supplementary Information.*

Answer: Thank you very much for your advice. We have revised in the Supporting Information files. Specifically speaking, we provide the full search strategy and search terms for PubMed and Cochrane library in details (original search image). The following highlight parts have been added as the supplementary information.

PubMed

**#1** ((((((((((fat grafting) OR (lipograft)) OR (lipoinjection)) OR (lipotransfer)) OR (fat transfer)) OR (fat transplant)) OR (lipostructure)) OR (lipofilling)) OR (fat injection)) OR (lipomodeling)) OR (fat transplantation) Sort by: Most Recent

**#2** (eyelid) OR (periocular) Sort by: Most Recent

**#3** #1 AND #2

Cochrane

(fat grafting OR lipograft OR lipoinjection OR lipotransfer OR fat transfer OR fat transplant OR lipostructure OR lipofilling OR fat injection OR lipomodeling OR fat transplantation) in All Text AND (eyelid OR periocular) in All Text - (Word variations have been searched)

The date ranges for the database searches (before November 20, 2020)

4. *Please provide the date ranges for the database searches you performed.*

Answer: Thank you very much for your advice. Likewise, we have revised in the Supporting Information files. We provide the data ranges for the database searches (original search image). The following highlight parts have been added as the supplementary information.

PubMed

**#1** ((((((((((fat grafting) OR (lipograft)) OR (lipoinjection)) OR (lipotransfer)) OR (fat transfer)) OR (fat transplant)) OR (lipostructure)) OR (lipofilling)) OR (fat injection)) OR (lipomodeling)) OR (fat transplantation) Sort by: Most Recent

**#2** (eyelid) OR (periocular) Sort by: Most Recent

**#3** #1 AND #2

Cochrane

(fat grafting OR lipograft OR lipoinjection OR lipotransfer OR fat transfer OR fat transplant OR lipostructure OR lipofilling OR fat injection OR lipomodeling OR fat transplantation) in All Text AND (eyelid OR periocular) in All Text - (Word variations have been searched)

The date ranges for the database searches (before November 20, 2020)

5. *Please attach a Supplemental file of the results of the individual components of the quality assessment, not just the overall score, for each study included. Please also explain the reasons, and number of studies excluded for each reason, in the flow diagram. Thank you.*

Answer: Thank you very much for your advice and we agree with you. We have given the individual scores for each study included, which will provide studies a clear quality assessment. The revised edition can be found in the Supporting Information files. And the flow diagram has been done in the Figure files.

NR: not reported

6. *Thank you for stating the following in the Acknowledgments Section of your manuscript:*

*"This study was funded by National Natural Science Foundations of China (81773488, 81772096 and 81803289)* *We note that you have provided funding information that is not currently declared in your Funding Statement. However, funding information should not appear in the Acknowledgments section or other areas of your manuscript. We will only publish funding information present in the Funding Statement section of the online submission form.*

Answer: Thank you very much for your advice. We apologize for the confusion regarding the funding statement. We have revised our Funding Statement in the online submission form.

7. *In your Data Availability statement, you have not specified where the minimal data set underlying the results described in your manuscript can be found. PLOS defines a study's minimal data set as the underlying data used to reach the conclusions drawn in the manuscript and any additional data required to replicate the reported study findings in their entirety. All PLOS journals require that the minimal data set be made fully available. For more information about our data policy, please see* [*http://journals.plos.org/plosone/s/data-availability*](http://journals.plos.org/plosone/s/data-availability)*.*

Answer: Thank you very much for your advice. We have provided the minimal data set in the Supporting Information files. We are very supportive of the Data Availability statement. So, we provide the raw data as clearly as possible with the belief that this behavior will promote academic development.

8. *We note that Figure 2 in your submission contain map images which may be copyrighted. All PLOS content is published under the Creative Commons Attribution License (CC BY 4.0), which means that the manuscript, images, and Supporting Information files will be freely available online, and any third party is permitted to access, download, copy, distribute, and use these materials in any way, even commercially, with proper attribution. For these reasons, we cannot publish previously copyrighted maps or satellite images created using proprietary data, such as Google software (Google Maps, Street View, and Earth). For more information, see our copyright guidelines:* [*http://journals.plos.org/plosone/s/licenses-and-copyright*](http://journals.plos.org/plosone/s/licenses-and-copyright)*.*

Answer: Thank you very much for your advice. We apologize for the confusion made in the Figure 2. Without taking the copyright into full consideration, we use the map downloaded from the internet. We have made a new “geographic distribution” map by the software “Hiplot”. The new picture can be found in the Figure files.

*Reviewer #1: I have read with great interest the manuscript entitled "Efficacy, safety and complications of autologous fat grafting for rejuvenation of eyelids and periorbital area: A systematic review and meta-analysis". These are my comments to the authors.
1) In the abstract although the authors introduce the abbreviation AFG for autologous fat grafting, they go back to using autologous fat grafting and not AFG in their conclusion. Please check the manuscript and correct this minor error*

Answer: Thank you very much for your advice. We apologize for the mistake made in the manuscript. We have revised the error, undoubtedly, this is our mistake, thanks for reminding us.

*2) In the abstract in the conclusion section they report that "AFG might be....and reconstruction) the title of the manuscript refers to evaluation of AFG in rejuvenation, i think the connection of AFG to the reconstruction of the periorbital region is not appropriate. I advice to only use the periorbital rejuvenation and contour defects.*

Answer: Thank you very much for your advice and we totally agree with you. We apologize for the confusion made in the title. We have revised the title to “Efficacy, safety and complications of autologous fat grafting to the eyelids and periorbital area: A systematic review and meta-analysis”. Indications in the meta-analysis concern a lot of diseases such as aging eyelids, tear trough deformity and anophthalmic reconstruction. The former title cannot give an all-around description of the content.

*3)Line 56 the word isolation is misspelled as insolation*

Answer: Thank you very much for your advice. We apologize for the spelling mistake in the manuscript. We have revised the content. We shouldn’t have made such a simple grammatical mistake.

*4)In the exclusion criteria line 85-86 the authors refer to patients with a history of other eyelid surgery but then give an example of periorbital rejuvenation and "reconstruction" with hyaluronic acid. Hyaluronic acid is used for rejuvenation and contour correction not for reconstruction and it is not a surgical procedure. The authors should specify and make this exclusion criteria clear to the read as to what they mean.*

Answer: Thank you very much for your advice. And we apologize for the confusion made in the manuscript. We have deleted the inappropriate passage in the manuscript.

*5) Line 473 a citation is mentioned as INVALID CITATION. The authors should check the references for errors*

Answer: Thank you very much for your advice. We apologize for the mistake. We have revised in the reference part.

*6) The article is generally well written but minor reviewing of language is needed.*

Answer: Thank you very much for your advice. Owing to the limited writing skill, we cannot write paper like native speakers. So, we seek help from a professional scientific editing service. We have provided the Certi­ficate of English Language Editing in the Supporting Information files.

*Reviewer #2: thank you very much to give me the opportunity to review this article. the authors made a systematic review using international guidelines and it is correct. I think that the meta analysis is poor and did not support the conclusions. it is important to do a meta analysis with high methods to support the high quality of the journal and the topic treated.*

*i suggest to made a flow chart diagram of the review.*

Answer: Thank you very much for your advice and we have considered your comments carefully. Indeed, there were several limitations in this study. First, nearly all of the articles in this meta-analysis were case series, obviously, low quality. There was a lack of access to RCTs, on account of setting RCTs might be viewed as unnecessary or unethical. So, we conducted quality evaluation in accordance with the international scale, for example, the Ottawa-Newcastle Scale, to evaluate the included literatures more objectively. Besides, we used a blinded method to ensure quality and a third reviewer resolved any disagreements. Second, the follow-up period seemed to present a lot of variability. According to already published theory, it will take 3 to 6 months for graft to reach a steady state. Thus, the studies included are followed over 3 months. Third, there was still debate about objective standards which could assess the validity of included studies. Studies included use a lot of objective scales, such as, 5-point Likert scale, grading scale, Fitzpatrick scale and so on. And we have made a flow chart diagram of the review in the “figure” file.

We thank you for your time and consideration and hope that the modified manuscript is now suitable for publication.

Sincerely yours,

Fan Yang

Department of Plastic Surgery and Burns,

Fourth Military Medical University,

Xi’an 710032, Shaanxi, China

Email: [crystalyangfan@163.com](mailto:crystalyangfan@163.com)
