# Supplementary material for: Efficacy, safety and complications of autologous fat grafting to the eyelids and periorbital area: A systematic review and meta-analysis
Source: PLoS One. 2021 Apr 1;16(4):e0248505. doi: 10.1371/journal.pone.0248505 (PMC8016360; doi:10.1371/journal.pone.0248505)
Supplement: Attachment — Submitted filename: Response to Reviewers.docx [file pone.0248505.s003.docx]

Dear Editors and Reviewers:

Thank you for your letter and for the reviewers’ comments concerning our manuscript entitled “Efficacy, safety and complications of autologous fat grafting to the eyelids and periorbital area: A systematic review and meta-analysis” (ID: PONE-D-20-28837R1). Those comments are all valuable and very helpful for revising and improving our paper, as well as the important guiding significance to our research. We have studied comments carefully and have made corrections which we hope to be met with approval. And we have uploaded the protocol to the protocols.io according to your requirements. Revised portion are marked with track changes in the paper. The main corrections in the paper are as follows:

*Editor: Please provide the date ranges for the database searches you performed in the main file of the manuscript (Methods section - Search strategy) and states if any language restriction was applied in the search process.*

Response: We are very sorry for our negligence of data range and language restriction. The specific information has been added to the methods section of the article.

A systematic database search was carried out before November 11, 2020. There were no restrictions with respect to language.

Thank you and best regards.

Yours sincerely,

Weilu Zhang

Email: [zhangweilu@126.com](mailto:zhangweilu@126.com)
